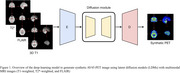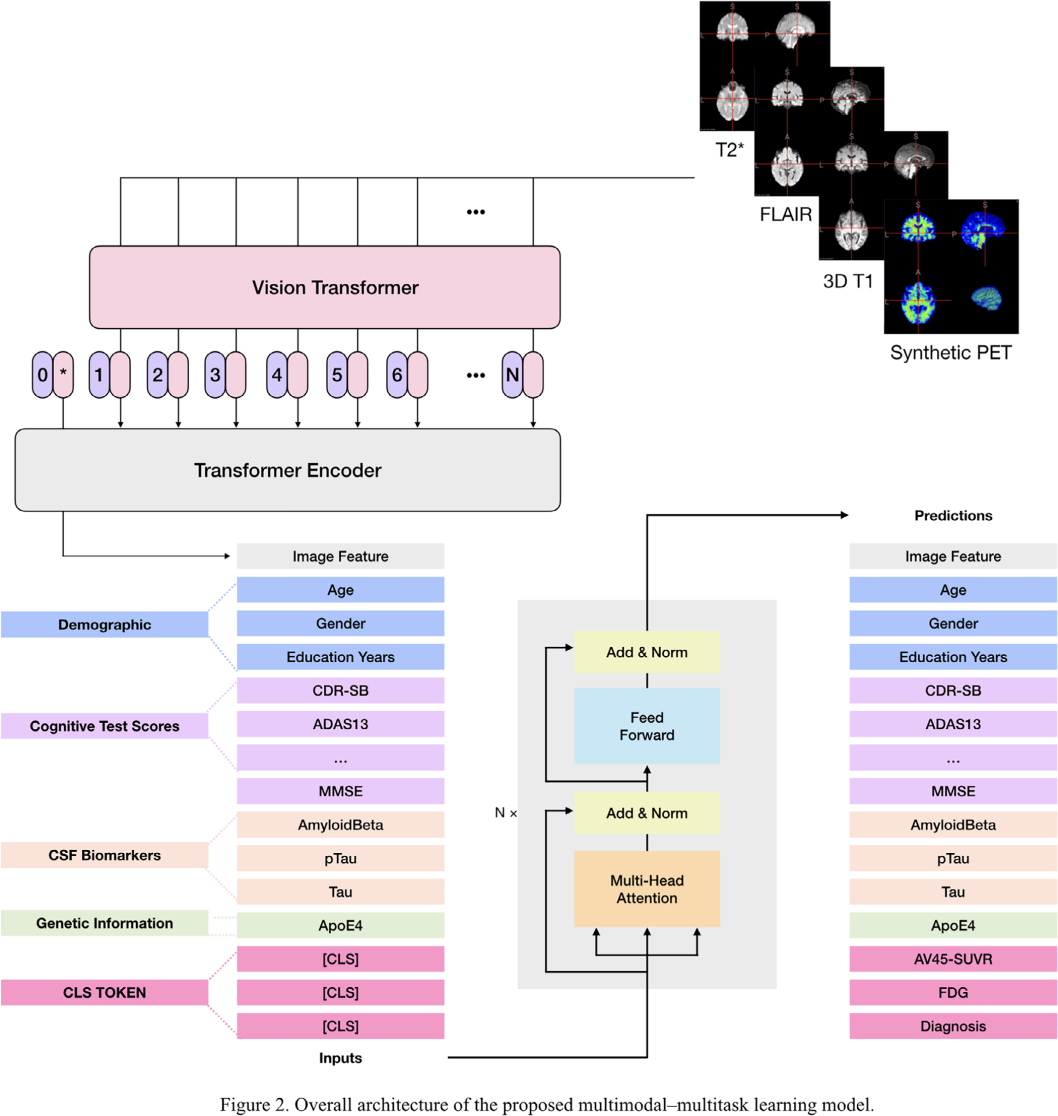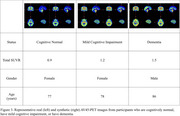# Predicting Amyloid Burden Using a Masked Multimodal‐Multitask Deep Learning Framework with Latent Diffusion‐based Synthetic PET

**DOI:** 10.1002/alz70862_110185

**Published:** 2025-12-23

**Authors:** Seungjun Lee, Wooseok Jung, Seung Hyun Lee, Geon‐Ho Jahng

**Affiliations:** ^1^ VUNO Inc., Seocho‐gu, Seoul Korea, Republic of (South); ^2^ Kyung Hee University Hospital at Gangdong, Seoul, Seoul Korea, Republic of (South); ^3^ Kyung Hee University, Seoul Korea, Republic of (South)

## Abstract

**Background:**

Alzheimer’s disease (AD) is characterized by pathological amyloid‐β accumulation. Positron emission tomography (PET) imaging remains a widely accepted gold standard for quantifying amyloid burden via standardized uptake value ratios (SUVRs); however, its prohibitive cost and limited availability restrict broader clinical adoption. Although recent deep learning models show promise in predicting amyloid burden, they often require complete clinical data or actual PET images—conditions rarely satisfied in routine clinical practice. To overcome these barriers, we propose a masked multimodal–multitask deep learning framework that integrates synthetic PET scans to improve amyloid burden prediction under real‐world data constraints.

**Method:**

We analyzed 2,043 longitudinal observations from 968 ADNI‐2 and ADNI‐3 participants (mean age: 72 years; male/female: 491/477). Each observation included T1‐weighted, T2*‐weighted, and FLAIR MRI, AV45‐PET, demographic data, APOE4 status, cognitive assessments, and CSF biomarkers. Our approach involved two main steps:

1. Synthetic PET generation: A latent diffusion model (LDM) was trained to generate synthetic AV45‐PET scans from MRI sequences.

2. Amyloid burden prediction: A deep learning network integrated these synthetic PET images with available clinical data through a masked embedding attention mechanism to explicitly handle missing inputs and predict both continuous amyloid SUVRs and masked clinical features.

We compared our method against three baselines: (i) MRI‐only, (ii) non‐imaging–only, and (iii) a multimodal model without synthetic PET. Performance on a held‐out test set was evaluated using mean absolute error (MAE) for SUVR prediction and area under the curve (AUC) for amyloid positivity (SUVR > 1.11).

**Result:**

For SUVR prediction, the baseline models achieved MAEs of 0.20, 0.13, and 0.13, whereas our approach reached 0.11. For amyloid positivity classification, the baselines yielded AUCs of 0.48, 0.89, and 0.90, while our model attained 0.93.

**Conclusion:**

By reducing reliance on cost‐intensive PET scans and handling missing data more effectively, our framework could substantially broaden access to early AD screening in diverse clinical settings. Future work will involve testing in larger, more varied cohorts, applying different tracers, and further evaluate its real‐world impact on AD management.